# Reliability and diagnostic performance of an automated MRI-based classifier compared with radiologists in Alzheimer’s disease

**DOI:** 10.3389/fninf.2026.1821249

**Published:** 2026-05-13

**Authors:** Nurmakhan Zholshybek, Elnora Abdurakhmanova, Almas Bimakhan, Dinara Jumadilova, Zhanibek Baiturlin, Joseph Almazan, Srinivasa Rao Bolla

**Affiliations:** 1 Department of Medicine, School of Medicine, Nazarbayev University, Astana, Kazakhstan; 2 Department of Biomedical Sciences, School of Medicine, Nazarbayev University, Astana, Kazakhstan; 3 Clinical and Academic Department of Radiology and Nuclear Medicine, Corporate Fund “University Medical Center”, Astana, Kazakhstan; 4 Department of Radiology and Radiosurgery, National Center for Neurosurgery, Astana, Kazakhstan

**Keywords:** Alzheimer’s disease, artificial intelligence, brain MRI, cognitive disorders, diagnostic accuracy

## Abstract

Reliable imaging biomarkers are essential for improving early detection of Alzheimer’s disease (AD). We evaluated whether an automated MRI-based classifier provides diagnostic performance comparable to expert radiologists in differentiating cognitively normal (CN) individuals from patients with AD using standardized ADNI data. Thirty-eight structural MRI datasets (20 CN, 18 AD) were analyzed. An automated multi-class volumetric classifier and two board-certified radiologists independently assigned probability scores across seven diagnostic categories. Performance was evaluated using a partial-credit scoring rule to account for probabilistic ties. Diagnostic performance for CN-AD discrimination was assessed using accuracy, sensitivity, specificity, receiver operating characteristic (ROC) analysis, inter-observer agreement metrics, Brier scores for calibration, and decision curve analysis (DCA) for clinical utility. The automated classifier achieved an accuracy of 0.66, sensitivity of 0.56, and specificity of 0.75. Radiologists demonstrated comparable performance with inherent inter-observer variability. Agreement between automated and human assessments was fair at the categorical level, with low concordance for continuous probability estimates. ROC analysis based on continuous AD probabilities demonstrated high discrimination performance for the automated model (AUC = 0.90), exceeding that of radiologists (AUC = 0.71 and 0.62). DCA indicated that the automated pipeline provides a positive net benefit as a second-opinion tool. This exploratory study emphasizes the impact of evaluation frameworks on performance metrics and supports further validation using multi-modal data in larger cohorts.

## 1 Introduction

Alzheimer’s disease (AD) and related dementias constitute an increasing share of the global neurodegenerative disease burden (Wang et al., 2024). According to the Global Burden of Disease 2021 estimates, dementia, with AD as its most prevalent subtype, has become a leading cause of disability and dependence among older adults, with a marked rise in the number of affected individuals worldwide over recent decades (Steinmetz et al., 2024). The etiology of most sporadic AD cases remains incompletely understood; however, longitudinal studies have demonstrated that reduced sleep duration and impaired sleep quality are associated with an increased risk of dementia, particularly AD, over long-term follow-up (Hahn et al., 2014; Ju et al., 2014).

Neurodegenerative processes in AD are characterized by progressive loss of brain tissue, which on magnetic resonance imaging (MRI) typically manifests as atrophy of the medial temporal lobe and associated enlargement of cerebrospinal fluid spaces (Teipel et al., 2013). In this context, automated quantitative analysis of brain MRI has significant potential to support objective diagnosis and longitudinal assessment of neurological diseases (Leandrou et al., 2018; Marek et al., 2025). MRI-based brain volumetry is increasingly applied to characterize disease-related structural changes and their progression. volBrain is an open-access, AI-powered platform for brain image analysis that provides automated pipelines for the volumetric assessment of multiple brain structures across a range of neurological conditions (Manjón and Coupé, 2016).

The cognitiveTree pipeline of volBrain is based on the concept of a lifespan tree of brain anatomy, modeling structural brain changes across the lifespan to characterize deviations from normal aging using MRI data (de Senneville et al., 2020). Its framework was developed using a large multi-center dataset comprising 37,594 T1-weighted MRI scans collected from 20 open-access databases, predominantly including cognitively normal aging (CN) individuals alongside multiple neurodegenerative conditions such as AD, dementia with Lewy bodies (DLB), behavioral variant frontotemporal dementia (bvFTD), semantic dementia (SD), progressive nonfluent aphasia (PNFA), and progressive supranuclear palsy (PSP). Model generalization was further validated on an independent external dataset of 1,754 subjects from six cohorts, including CN, AD, and other neurodegenerative disorders (Coupé et al., 2025).

**FIGURE 1:**
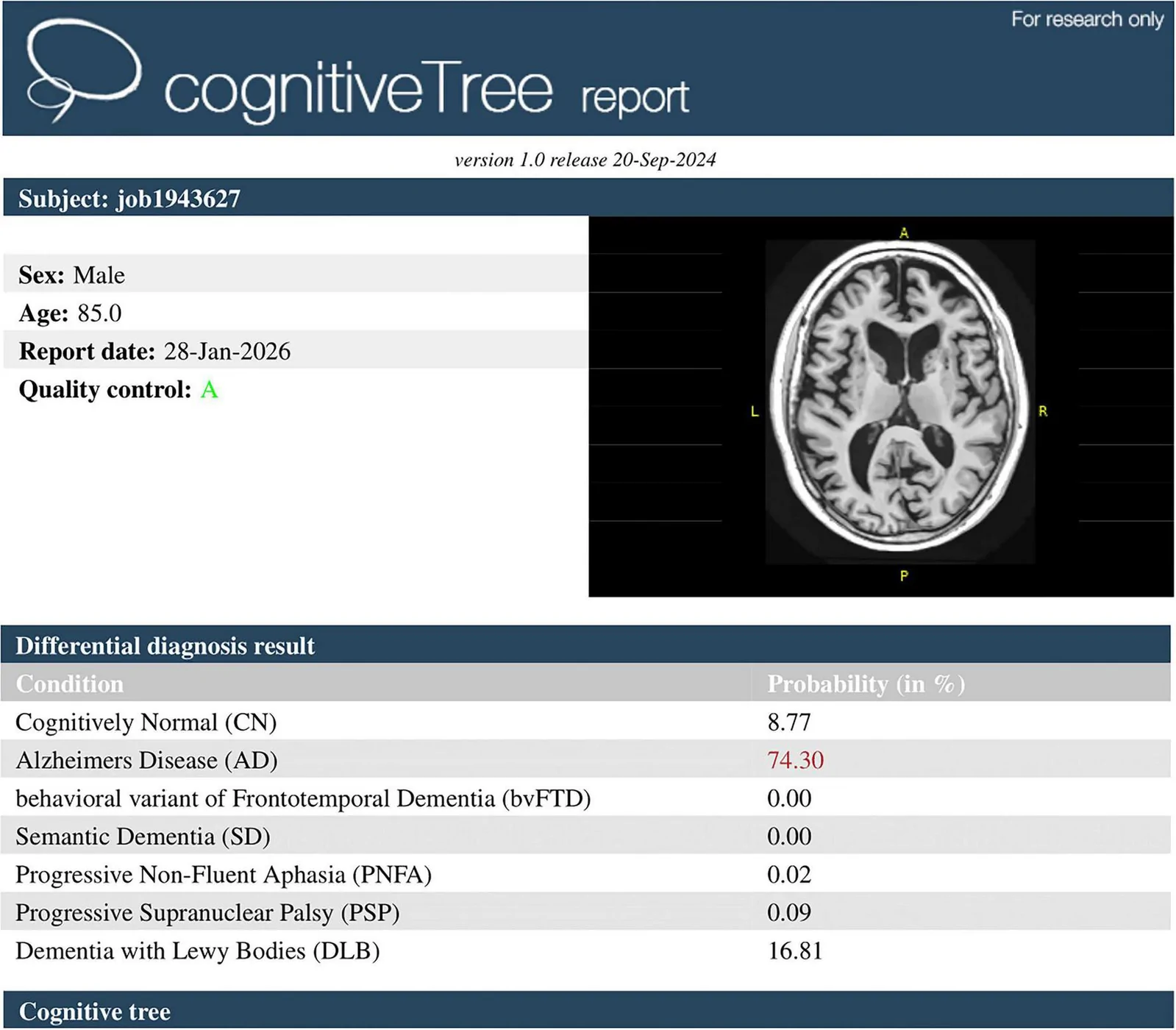
The cognitiveTree report presents a differential diagnosis across seven cognitive conditions (cognitively normal aging, Alzheimer’s disease (AD), behavioral variant frontotemporal dementia, semantic dementia, progressive nonfluent aphasia, progressive supranuclear palsy, and dementia with Lewy bodies), with estimated probability scores expressed as percentages. In this example, obtained from an 85-year-old male subject, the estimated probability for AD is 74.3%.

The automated pipeline operates as a multi-class neuroimaging classifier for discriminating among CN, AD, bvFTD, SD, PNFA, PSP, and DLB (Coupé et al., 2025). For each subject, the model outputs probability scores corresponding to these diagnostic categories, reflecting proximity to disease-specific branches of the lifespan tree (Figure 1). In addition, the pipeline provides maps of brain structures deviating from normative aging patterns, highlighting anatomical regions that contribute to the final classification (Coupé et al., 2020). Although cognitiveTree has been developed and validated as a multi-class classifier across multiple neurodegenerative conditions, its performance in focused binary clinical scenarios such as CN versus AD remains less well characterized. A significant research gap exists in understanding how multi-class automated systems perform relative to human experts when evaluating diagnostic uncertainty through a shared probabilistic framework. This exploratory study aims to compare the diagnostic performance of cognitiveTree and radiologists in differentiating CN and AD. We hypothesize that the automated classifier will demonstrate high discriminatory performance and complement radiologists’ assessment, potentially reducing clinical workload by serving as a reliability anchor for probabilistic outputs.

## 2 Related works

The evolution of imaging biomarkers for AD has transitioned from labor-intensive manual measurements to sophisticated automated pipelines, driven by the global need for objective and scalable diagnostic tools (Chedid et al., 2022; Shukla et al., 2023). Early research in quantitative MRI focused predominantly on the medial temporal lobe, specifically hippocampal atrophy, as a primary hallmark of neurodegeneration (Schröder and Pantel, 2016; van de Pol et al., 2006). While initial software solutions provided basic automated segmentations, recent advancements have introduced deep learning architectures capable of precise analysis. For instance, the volBrain platform (Manjón and Coupé, 2016) utilizes an ensemble of convolutional neural networks known as AssemblyNet to segment over one hundred brain structures simultaneously, demonstrating significant robustness across diverse imaging sources and clinical populations (Jytzler and Lysdahlgaard, 2024).

Beyond simple volumetry, a substantial body of literature now explores the use of machine learning and deep learning for the automated classification of AD. Recent studies (Aslan and Özüpak, 2025; Aslan et al., 2025; Firat and Üzen, 2024) have successfully implemented diverse models, ranging from standard convolutional neural networks to advanced transformer-based architectures, to differentiate between CN individuals and those with AD. The cognitiveTree framework builds upon these technical foundations by modeling structural brain changes across the human lifespan. By characterizing deviations from normative aging patterns, this multi-class system allows for the differentiation of various neurodegenerative conditions that often present with overlapping anatomical features (Coupé et al., 2025).

Despite the high accuracy reported for these AI systems under controlled experimental conditions, the comparison between automated performance and human expert assessment remains a critical area of investigation. Research indicates that human radiologists and AI models often utilize fundamentally different decision strategies (McLeod et al., 2026; Sorantin et al., 2022). While automated models are primarily driven by objective anatomical pattern similarity (Marquand et al., 2013), human readers typically integrate clinical heuristics and contextual considerations into their visual assessments (Castillo et al., 2021; Li et al., 2022; Patel and Itri, 2022). While some systems have approached neuroradiologist-level accuracy in differential diagnosis (Rauschecker et al., 2020), there is a recognized need to better understand how these two “observers” manage diagnostic uncertainty and probabilistic outputs. Current evidence suggests that rather than serving as a replacement for human expertise, AI may be most effective when integrated into a collaborative clinical workflow where it provides quantitative support to supplement the radiologist’s contextual interpretation (Agarwal et al., 2023).

## 3 Materials and methods

### 3.1 Materials

Data used in the preparation of this article were obtained from the Alzheimer’s Disease Neuroimaging Initiative (ADNI) database (adni.loni.usc.edu). The ADNI was launched in 2003 as a public-private partnership, led by Principal Investigator Michael W. Weiner, MD. The original goal of ADNI was to test whether serial magnetic resonance imaging (MRI), positron emission tomography (PET), other biological markers, and clinical and neuropsychological assessment can be combined to measure the progression of mild cognitive impairment (MCI) and early Alzheimer’s disease (AD). The current goals include validating biomarkers for clinical trials, improving the generalizability of ADNI data by increasing diversity in the participant cohort, and to provide data concerning the diagnosis and progression of Alzheimer’s disease to the scientific community. For up-to-date information, see adni.loni.usc.edu.

The study obtained approval as an exemption from the Nazarbayev University Institutional Research Ethics Committee (NU-IREC) because it used an open ADNI dataset. The approval number: 1183/27012026.

Subjects were selected from the ADNI-1 cohort based on specific criteria with a baseline 3D T1-weighted structural MRI scan at 3T of a subject older than 45 years and available age and sex metadata. Exclusion criteria included the presence of significant focal brain lesions, non-AD neurodegenerative pathologies, or poor image quality that could impede automated segmentation. To minimize representation bias and ensure balanced group comparisons, 38 anonymized structural MRI datasets, including 20 cases of CN and 18 cases of AD, were selected.

Ground truth labels were established using standardized ADNI clinical protocols. CN status was defined by Mini-Mental State Examination (MMSE) scores of 24-30 and a Clinical Dementia Rating (CDR) of 0, while AD patients met the NINCDS/ADRDA Alzheimer’s criteria for probable AD with MMSE scores of 20-26 and a CDR of 0.5 or 1.0. These labels were corroborated by longitudinal assessments and biomarker data provided by ADNI to ensure diagnostic accuracy. Age and sex were provided to both the model and radiologists to account for demographic variation. This sample size was chosen to balance the feasibility of intensive, multi-class probabilistic scoring by experts with the requirements for an exploratory performance comparison. A power analysis for the comparison of two correlated areas under the ROC curve (AUC) indicates that with 38 subjects and an assumed moderate correlation (*r* = 0.50) between observers, the study is powered at 80% (α = 0.05) to detect a difference in AUC of 0.21.

### 3.2 Model description and diagnostic targets

The cognitiveTree model was developed to classify individuals across seven diagnostic categories: CN, AD, bvFTD, SD, PNFA, PSP, and DLB. While the model outputs probability scores for all seven classes, the present study focused on the discrimination between CN and AD due to their high prevalence and clinical relevance in neurodegenerative practice. This approach was further supported by the availability of high-confidence ground truth labels for CN and AD within the ADNI database. To validate this focused evaluation, the predicted diagnosis was defined as the category with the highest assigned probability among all seven available classes. Any case where the model assigned the highest probability to a non-AD/CN category was treated as a misclassification for the binary outcome. This ensured that the multi-class nature of the tool was preserved while rigorously assessing its performance in the most common clinical scenario.

### 3.3 Training and output

**FIGURE 2:**
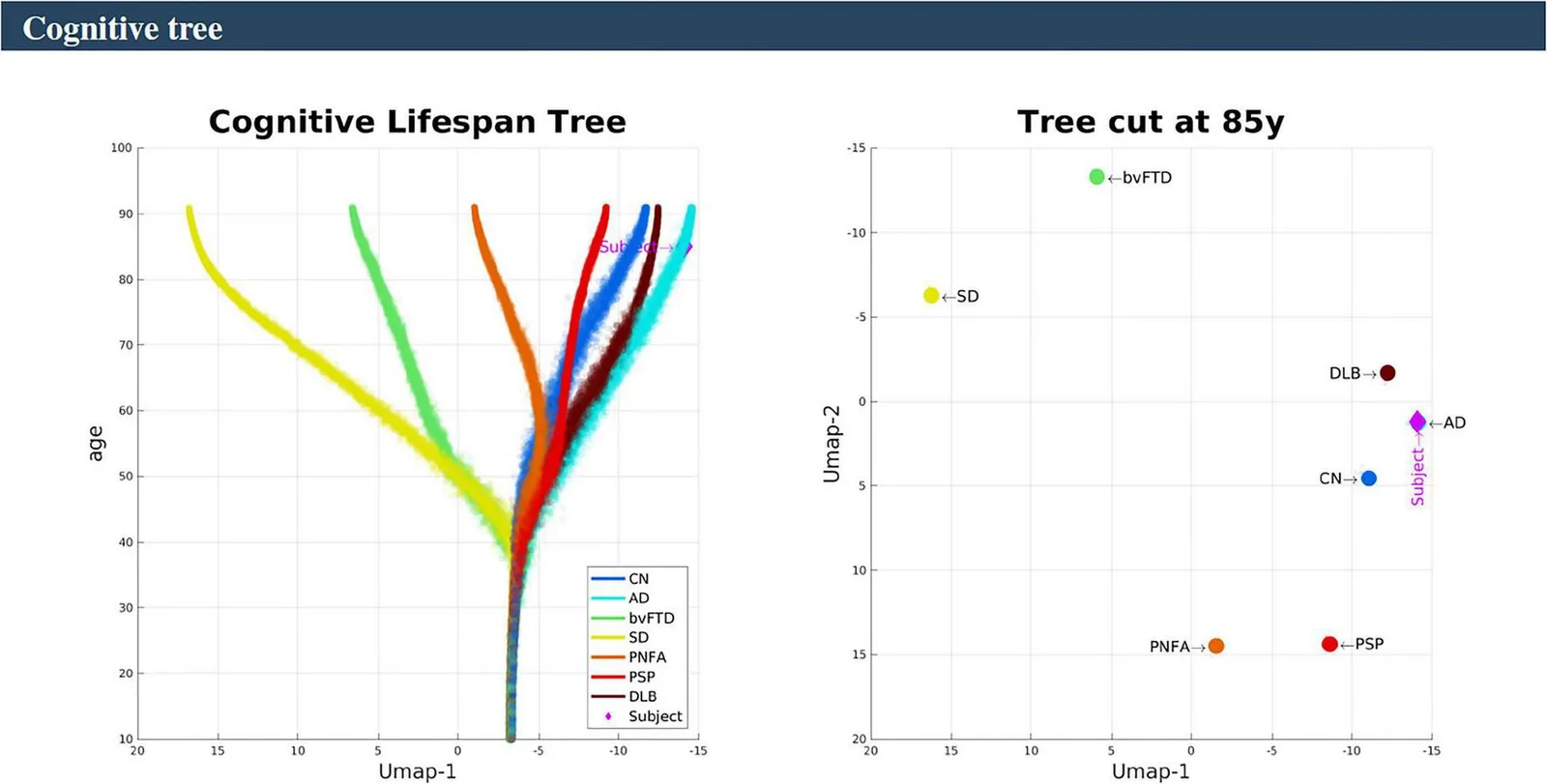
The cognitiveTree report also includes a 3D cognitive lifespan tree (left) composed of seven branches corresponding to cognitive conditions (CN, cognitively normal; AD, Alzheimer’s disease; bvFTD, behavioral variant frontotemporal dementia; SD, semantic dementia; PNFA, progressive nonfluent aphasia; PSP, progressive supranuclear palsy; DLB, dementia with Lewy bodies), with a 2D cross-section tree cut at a specific age (right). In this example, obtained from an 85-year-old male subject, the estimated probability for AD is 74.3%.

cognitiveTree is an externally developed and validated tool; full details of model architecture, training procedures, and hyperparameters are reported in the primary reference (Coupé et al., 2025). The pipeline performs whole-brain segmentation using AssemblyNet, a convolutional neural network ensemble that generates volumetric measurements for 133 brain structures (Coupé et al., 2020). Volumetric measurements are normalised for age and sex prior to classification. For each subject, the model produces probability scores, one for each of the seven diagnostic categories, summing to 100%, representing the subject’s structural proximity to each disease-specific branch of a lifespan tree (Figure 2). The pipeline was applied without modification to model parameters.

### 3.4 Radiologists’ analysis

**FIGURE 3:**
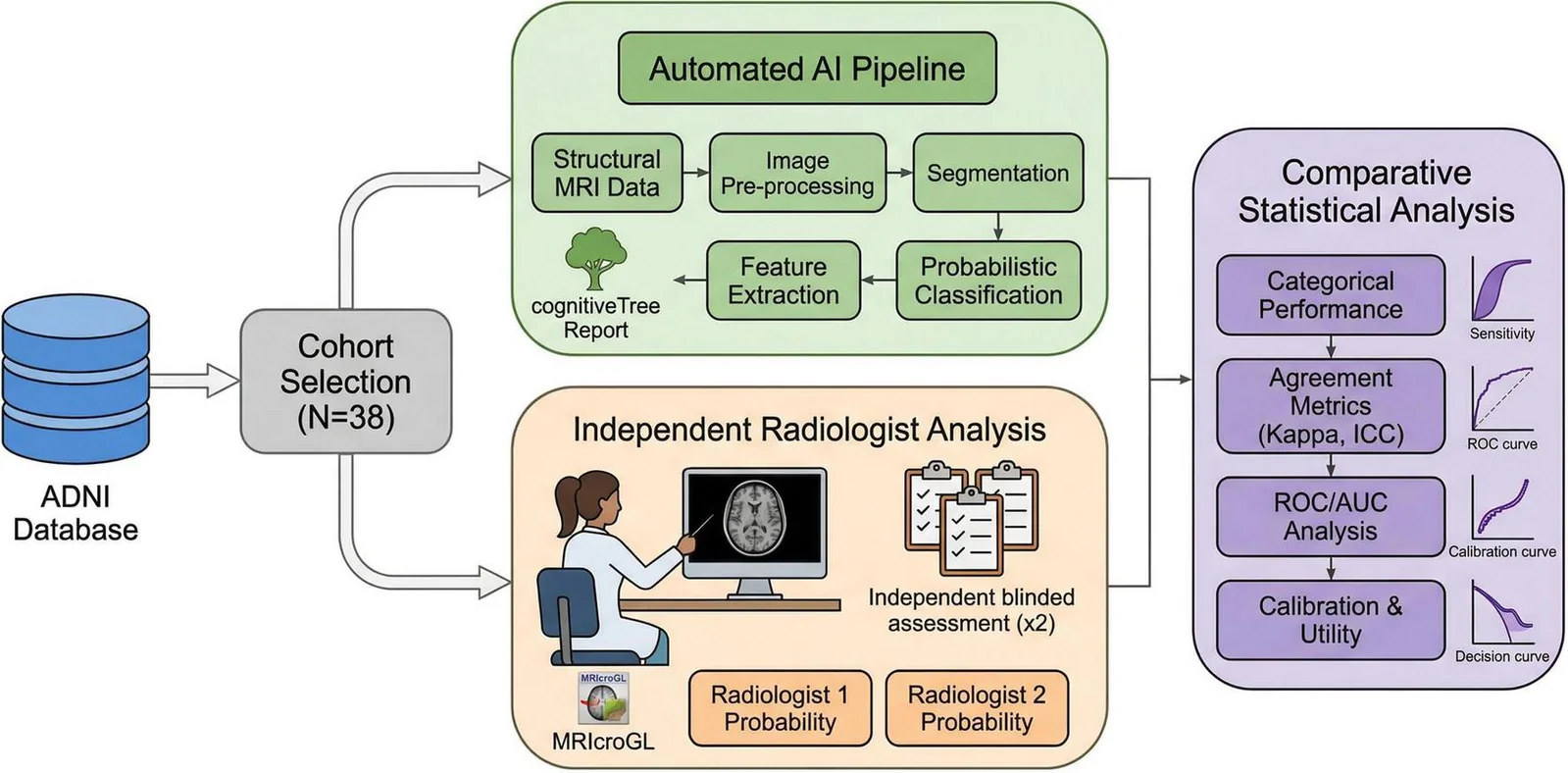
Study workflow and analytical pipeline. Schematic representation of the study design, including: data selection from the ADNI database; automated structural MRI processing via the cognitiveTree pipeline; independent, blinded probability assignments by two board-certified radiologists; and comparative statistical analysis using categorical performance, agreement, ROC/AUC, and calibration and utility metrics.

Two board-certified radiologists independently reviewed a set of 38 structural brain MRI examinations provided in NIfTI format (.nii.gz) and the subject’s age and sex. All scans were evaluated using MRIcroGL software (version 1.2.20220720) (Rorden, 2025). Radiologists were instructed to assign probability scores across the same seven diagnostic categories as cognitiveTree (CN, AD, bvFTD, SD, PNFA, PSP, and DLB). This probabilistic approach was intentionally adopted to enable direct, quantitative head-to-head comparison with the model’s continuous outputs. While it differs from routine clinical reporting, where radiologists typically provide descriptive diagnostic impressions, the use of subjective probability assignments is a validated method for characterizing diagnostic uncertainty and evaluating observer performance. This protocol allows for the application of advanced statistical metrics, such as ROC analysis on a continuous scale, providing a more granular assessment of diagnostic reasoning than binary forced-choice tasks. Radiologists performed their assessments independently and were blinded to the outputs of cognitiveTree and to each other’s evaluations. The overall study workflow, from data acquisition and automated processing to independent radiologist assessment and statistical validation, is illustrated in Figure 3.

The predicted diagnosis was defined as the category with the highest assigned probability among all seven classes. Diagnostic correctness was scored as fully correct when the true diagnosis uniquely had the highest probability, incorrect when another category had the highest probability, and partially correct only in cases where multiple categories shared the highest probability, with credit divided equally among tied categories.

### 3.5 Statistical analysis

Statistical analyses were performed to assess diagnostic performance, agreement, and variability between cognitiveTree and radiologist probability outputs. Continuous variables were summarized as mean ± standard deviation. All analyses were two-tailed, and a *p* < 0.05 was considered statistically significant.

For diagnostic classification, the predicted label was defined as the diagnostic category with the highest assigned probability among seven cognitive conditions (CN, AD, bvFTD, SD, PNFA, PSP, and DLB). In cases where two or three diagnostic categories shared the highest probability, partial-credit scoring was applied, with credit divided equally among tied categories if the true diagnosis was included among them; otherwise, the case was scored as incorrect.

Diagnostic performance for differentiating AD from CN was quantified using accuracy, sensitivity, and specificity, with 95% confidence intervals (CI) calculated using the Wilson method. Weighted F1-scores were computed to account for class imbalance. Categorical agreement between observers was assessed using Cohen’s κ, interpreted according to the Landis and Koch scale (Landis and Koch, 1977).

The relationship between continuous AD probability scores generated by cognitiveTree and radiologists was evaluated using Pearson’s correlation coefficient for normally distributed data and Spearman’s rank correlation coefficient when normality assumptions were not met. Agreement in probability estimates was further examined using the intraclass correlation coefficient (ICC, two-way mixed-effects model, absolute agreement).

Systematic bias and limits of agreement between probability estimates were visualized using Bland-Altman plots. Discriminatory performance for differentiating AD from CN was assessed using receiver operating characteristic (ROC) curve analysis, and the area under the curve (AUC) was calculated for each observer. To quantify the precision of these estimates, 95% CIs for AUC were determined using a bootstrap method with 2,000 iterations. ROC analysis was performed using continuous AD probability scores as the predictor variable, rather than categorical class assignments, to evaluate ranking performance across all thresholds. Differences between AUCs were evaluated using DeLong’s test. Beyond discrimination, the reliability of the probabilistic outputs was evaluated through a calibration analysis. This included the calculation of Brier scores, where a score closer to zero indicates superior alignment between predicted probabilities and actual outcomes, and the generation of calibration curves. To assess the clinical utility of the model, we performed a decision curve analysis (DCA) to calculate the “net benefit” of using cognitiveTree-guided diagnosis across a range of threshold probabilities (P_*t*_) in comparison to standard “diagnose all” and “diagnose none” strategies. Variability in probability distributions between observers was examined using Levene’s test.

To characterize the nature of cognitiveTree classification errors, the highest-probability category assignment was determined for each case and tabulated across all seven diagnostic categories stratified by true diagnostic label. Mean cognitiveTree AD probability scores were calculated separately for true AD and true CN cases to assess the direction and magnitude of any systematic difference in AD probability ranking between groups.

Statistical computations were performed using a combination of computational tools. GraphPad Prism (version 10.0; GraphPad Software, San Diego, CA, United States) was used for data visualization, including ROC curves and Bland–Altman plots. R software (version 4.3.2) was used for statistical calculations, including correlation analysis, intraclass correlation coefficient estimation, bootstrapped confidence intervals, Brier score calculation, and DCA. Analytical workflows were cross-checked to ensure consistency of results.

## 4 Results

A total of 38 structural MRI examinations were analyzed, including 18 patients with AD and 20 CN individuals. Detailed demographic and clinical characteristics of the study cohort, including age and sex distribution stratified by diagnostic group, are summarized in Table 1. Probability scores across seven diagnostic categories (CN, AD, bvFTD, SD, PNFA, PSP, and DLB) were obtained from cognitiveTree and two radiologists for all cases. Continuous variables were summarized as mean ± standard deviation where applicable.

**TABLE 1:** Demographic characteristics of the study cohort.

Characteristic	Total (*n* = 38)	AD (*n* = 18)	CN (*n* = 20)	*p*-value
Age (years), mean ± SD	75.3 ± 7.2	75.9 ± 7.9	74.8 ± 6.6	0.63
Sex (Male/Female)	18/20	9 / 9	9/11	0.82

### 4.1 Correlation analysis

Correlation analysis of AD probability scores demonstrated no statistically significant association between cognitiveTree and Radiologist 1 (Pearson *r* = 0.16, *p* = 0.35; Spearman ρ = −0.28, *p* = 0.08). Similarly, no significant correlation was observed between cognitiveTree and Radiologist 2 (Pearson *r* = −0.05, *p* = 0.76; Spearman ρ = 0.22, *p* = 0.18).

### 4.2 Agreement analysis

Inter-observer agreement for AD probability estimates, assessed using a two-way mixed-effects intraclass correlation coefficient (ICC -3.1, absolute agreement), was −0.22 between cognitiveTree and Radiologist 1 and −0.37 between cognitiveTree and Radiologist 2. Categorical agreement for CN-AD classification, evaluated using Cohen’s κ with tie-aware scoring, was 0.21 for cognitiveTree versus Radiologist 1 and 0.26 for cognitiveTree versus Radiologist 2, corresponding to fair agreement.

### 4.3 Bland-Altman analysis

**FIGURE 4:**
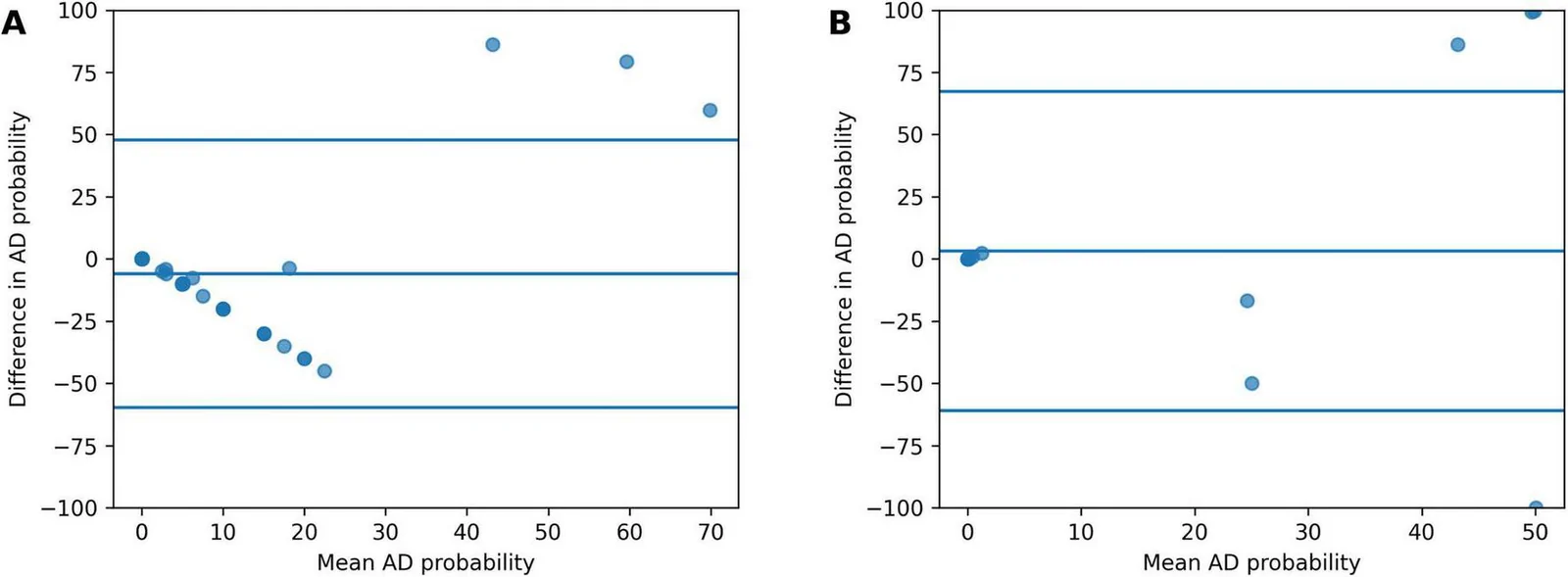
Bland-Altman plots demonstrating the mean Alzheimer’s disease (AD) probability on the x-axis and the difference in AD probability on the y-axis, with the mean difference and 95 percent limits of agreement between cognitiveTree and Radiologist 1 **(A)** and between cognitiveTree and Radiologist 2 **(B)**.

Bland-Altman analysis was performed to assess agreement between cognitiveTree and radiologists for continuous AD probability estimates. For cognitiveTree versus Radiologist 1, the mean difference in AD probability scores was −0.08, with 95% limits of agreement ranging from −0.42 to 0.26 (Figure 4A). For cognitiveTree versus Radiologist 2, the mean difference was −0.05, with 95% limits of agreement between −0.47 and 0.37 (Figure 4B). No systematic trend in differences across the range of mean probability values was observed.

### 4.4 Diagnostic performance

Using the highest-probability decision rule with partial-credit scoring applied only when multiple categories shared the highest probability, cognitiveTree achieved an accuracy of 0.66, sensitivity for AD of 0.56, and specificity for CN of 0.75. Radiologist 1 achieved an accuracy of 0.54, AD sensitivity of 0.36, and CN specificity of 0.70. Radiologist 2 achieved an accuracy of 0.60, AD sensitivity of 0.46, and CN specificity of 0.73. Weighted F1-scores were 0.71 for cognitiveTree, 0.63 for Radiologist 1, and 0.66 for Radiologist 2 (Table 2).

**TABLE 2:** Diagnostic performance comparison for CN-AD classification.

Readers	Accuracy (95% CI)	Sensitivity AD (95% CI)	Specificity CN (95% CI)	Weighted F1	Cohen’s κ
cognitiveTree	0.66 (0.51–0.81)	0.56 (0.40–0.72)	0.75 (0.61–0.89)	0.71	0.32
Radiologist 1	0.54 (0.38–0.69)	0.36 (0.20–0.52)	0.70 (0.54–0.86)	0.63	0.21
Radiologist 2	0.60 (0.44–0.75)	0.46 (0.29–0.63)	0.73 (0.57–0.89)	0.66	0.26

### 4.5 ROC analysis

**FIGURE 5:**
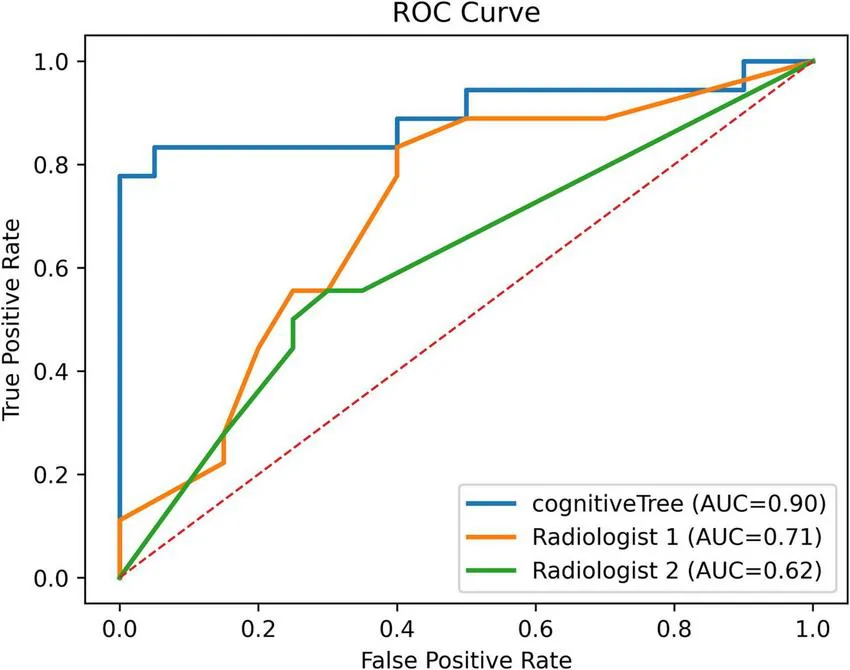
ROC curves for cognitiveTree, Radiologist 1, and Radiologist 2 in differentiating cognitively normal (CN) individuals from patients with Alzheimer’s disease (AD), derived from continuous AD probability scores assigned by each observer. The red dashed diagonal represents chance-level discrimination (AUC = 0.50). Radiologist 1 (orange) and Radiologist 2 (green) achieved AUC values of 0.71 and 0.62, respectively. The cognitiveTree model (blue) achieved an AUC of 0.90, indicating superior discrimination performance. The staircase appearance of the curves reflects the limited sample size. AUC differences were evaluated using DeLong’s test.

ROC analysis was performed using continuous AD probability scores generated by each observer as the ranking variable, with the ground-truth binary label (AD vs. CN) as the outcome. The AUC was 0.897 (95% CI: 0.764–1.000) for cognitiveTree, 0.710 (95% CI: 0.531–0.872) for Radiologist 1, and 0.617 (95% CI: 0.445–0.776) for Radiologist 2 (Figure 5).

### 4.6 Calibration and clinical utility

**FIGURE 6:**
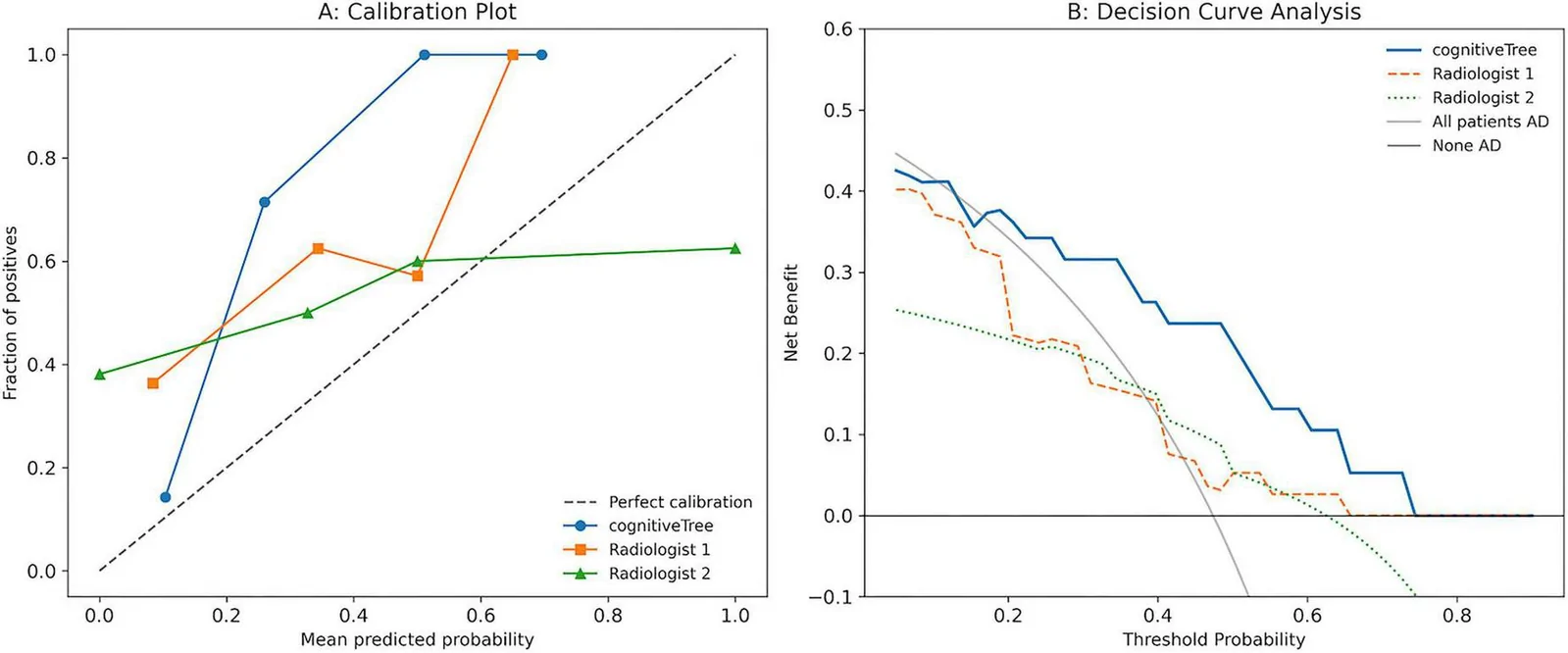
Calibration and decision curve analysis. Calibration plot showing the relationship between predicted AD probability and observed fraction of positives **(A)**: cognitiveTree (blue) demonstrates superior calibration (Brier = 0.192) compared to Radiologist 1 (orange) and Radiologist 2 (green). Decision curve analysis evaluating clinical utility **(B)**: cognitiveTree (solid blue) provides a higher net benefit across the clinically relevant threshold range compared to human experts and the “all patients AD” (gray) strategy.

Calibration analysis (Figure 6A) yielded a Brier score of 0.192 for cognitiveTree, indicating superior alignment between predicted probabilities and actual outcomes compared to Radiologist 1 (0.275) and Radiologist 2 (0.348). DCA (Figure 6B) demonstrated that using cognitiveTree to guide diagnostic decisions provided a positive net benefit across threshold probabilities from 0.1 to 0.9, consistently outperforming both human expert strategies and the default “all patients AD” strategy.

### 4.7 Variability analysis

Levene’s test showed no significant difference in variance between AD probability distributions generated by cognitiveTree and Radiologist 1 (*F* = 0.30, *p* = 0.59) or between cognitiveTree and Radiologist 2 (*F* = 0.39, *p* = 0.54).

### 4.8 Error analysis

**FIGURE 7:**
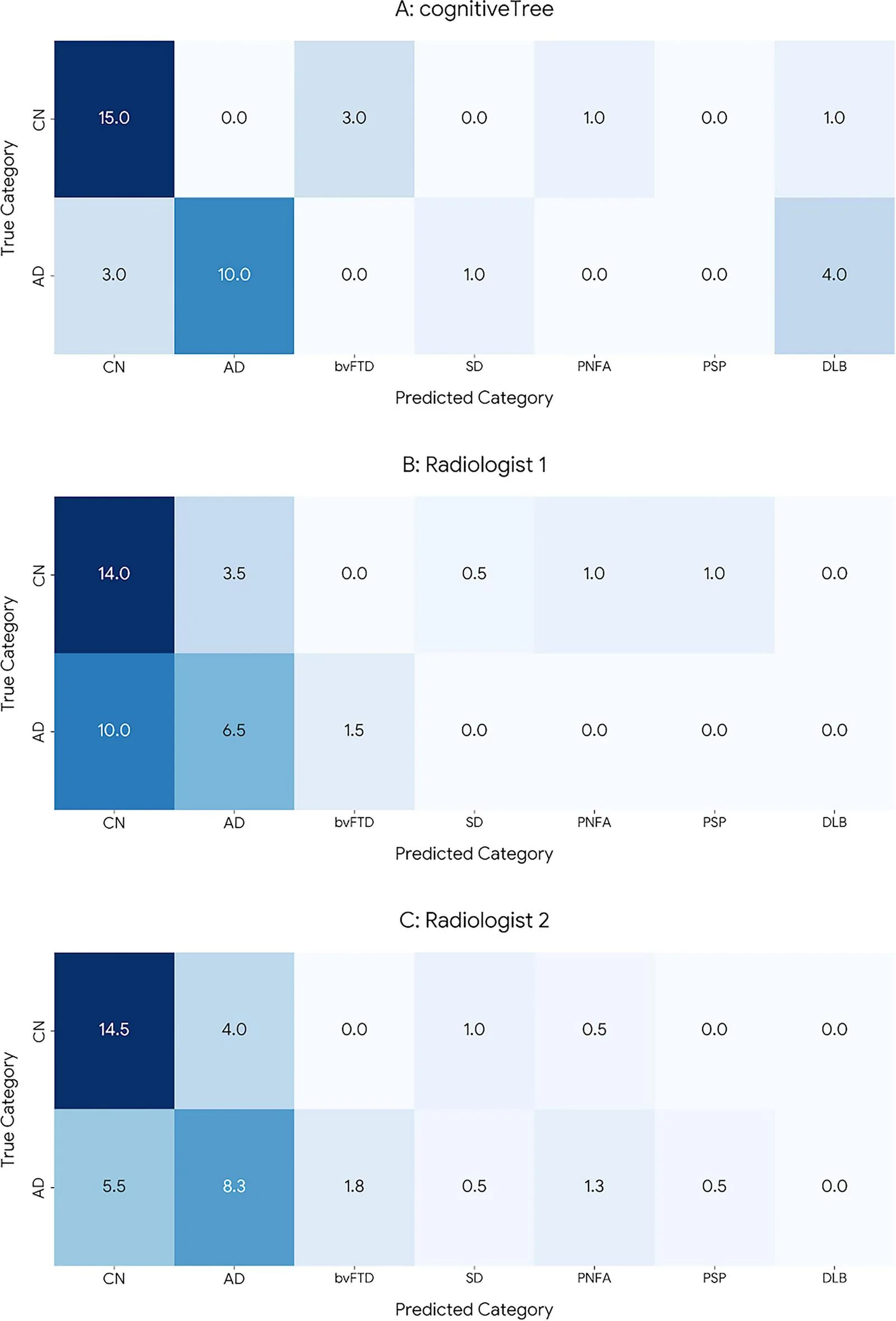
Multi-class confusion matrices with partial-credit scoring. Heatmaps displaying diagnostic performance for cognitiveTree **(A)**, Radiologist 1 **(B)**, and Radiologist 2 **(C)** across seven diagnostic categories (CN, cognitively normal; AD, Alzheimer’s disease; bvFTD, behavioral variant frontotemporal dementia; SD, semantic dementia; PNFA, progressive nonfluent aphasia; PSP, progressive supranuclear palsy; DLB, dementia with Lewy bodies). Rows represent ground-truth labels (CN and AD), and columns represent the predicted category based on the highest probability assignment. Labels for the primary categories of interest are bolded. Values reflect total case counts; decimal entries indicate instances where observers assigned tied highest probabilities, with the frequency distributed equally among the tied categories.

The distribution of the highest probability assignments across all seven diagnostic categories, accounting for diagnostic uncertainty through partial-credit scoring, is presented in Figure 7. The results confirm that the study cohort comprised 20 CN and 18 AD individuals. cognitiveTree demonstrated a distinct error profile by distributing alternative assignments across the multi-class spectrum, notably toward the DLB category (4 AD cases). In contrast, radiologists exhibited more frequent uncertainty between the CN and AD categories.

Mean cognitiveTree AD probability was 41.82% ± 22.17% in true AD cases and 11.54% ± 6.83% in true CN cases. In comparison, the mean cognitiveTree CN probability was 46.59% ± 23.71% in true CN cases and 17.64% ± 17.06% in true AD cases.

## 5 Discussion

This study compared the performance of an automated multi-class neuroimaging classifier with that of expert radiologists for differentiating CN and AD using structural MRI. The findings indicate that cognitiveTree and radiologists achieve broadly comparable performance while adopting different approaches to diagnostic reasoning and uncertainty.

The methodological choice to evaluate a multi-class classifier within a binary CN-AD framework mirrors the primary diagnostic challenge in clinical memory settings (Frisoni et al., 2009; Zhao et al., 2014). While cognitiveTree is designed for a broader differential diagnosis, focusing on AD and CN allows for a standardized comparison using the robust ground truth provided by ADNI. To ensure validity, we utilized a highest-probability decision rule across all seven diagnostic branches. This framework ensured that binary performance metrics were not artificially inflated, as the model was required to correctly distinguish AD not only from CN but also from other potential neurodegenerative patterns represented in its output.

A significant methodological innovation in this study was the adoption of a probabilistic evaluation protocol for radiologists (Halpern and Gazelle, 2003). While this departs from routine descriptive reporting, the use of explicit probability assignments was essential to enable a mathematically valid, head-to-head comparison with the automated model’s continuous outputs. This design is supported by previous research demonstrating that AI-human comparisons in neuroimaging are most granular when both “observers” are assessed on a continuous differential diagnosis scale (Psoter et al., 2014; Rauschecker et al., 2020; Rudie et al., 2020). By requiring probability distributions, we captured intermediate decision states and tied assignments, often lost in forced-choice studies that reflect the inherent heuristic uncertainty (Ranschaert et al., 2021) experts face in memory clinic settings.

Radiologists predominantly constrained their diagnostic hypotheses to CN and AD, rarely assigning the probability scores to alternative neurodegenerative conditions. This approach may increase specificity within a targeted clinical context but is frequently associated with expressions of uncertainty, often reflected as tied probability assignments. In contrast, cognitiveTree distributes probability across multiple diagnostic categories. The application of partial-credit scoring (Cohen, 1968) enabled a more nuanced representation of radiologist performance, capturing intermediate decision states that are not reflected in strict binary classification. Although radiologists demonstrated lower AUC values, their categorical accuracy was comparable to that of cognitiveTree, suggesting that performance differences depend on the evaluation metric applied.

ROC analysis demonstrated higher AUC values for cognitiveTree, indicating stronger ranking performance based on continuous probability outputs. However, this finding should be interpreted in the context of model design. As a multi-class system, cognitiveTree expresses AD probability relative to other diagnostic categories rather than as a binary classification. This may reduce apparent separability, particularly in cases where imaging features overlap, such as early AD and normal aging, or where atrophy patterns are heterogeneous (Byun et al., 2015; Poulakis et al., 2018). Therefore, differences between AUC, categorical performance metrics, and the relatively modest agreement are expected and reflect distinct aspects of model behavior.

Comparative analysis against traditional diagnostic benchmarks provides further context for these results. Prior research has established that automated volumetric assessments can offer diagnostic efficacy comparable to expert visual assessment in AD (Min et al., 2017). While volumetric MRI is recognized as a potent predictor of disease progression that often outperforms standard clinical predictors (Fleisher et al., 2008), the present data suggest that a multi-class Bayesian approach provides additional granularity by integrating these volumes into a probabilistic framework. This alignment with established AI-based diagnostic trends (Yao et al., 2023) reinforces the model’s utility as a high-performance tool for discriminating AD from normal aging.

Clinical translation of these results suggests that cognitiveTree may be useful as a supportive tool in memory clinic settings, particularly where the diagnosis is uncertain or atypical (Dickerson et al., 2017; Dubois et al., 2023; Ghezzi, 2018). For example, when imaging findings are not consistent with a typical AD pattern, multi-class probability outputs may suggest alternative diagnostic considerations and prompt further evaluation. In contrast, radiologists remain central to clinical decision-making, particularly in cases where focused binary differentiation is sufficient. An integrated approach, in which AI supports differential diagnosis and radiologists provide contextual interpretation, may therefore be beneficial.

Beyond diagnostic accuracy, the results underscore the necessity of aligning evaluation methods with model characteristics. Ensuring the generalizability of such models remains a challenge, as machine-learning tools often face reproducibility issues across diverse neuroimaging datasets (Aghdam et al., 2025). Variability analyses showed comparable dispersion of probability estimates, suggesting that observed differences are more likely related to decision strategy than to instability of outputs (Tourassi et al., 2013). The discrepancy between classification metrics and AUC arises from differences in evaluation frameworks: categorical metrics were based on a highest-probability decision rule across multiple classes, whereas ROC analysis evaluates continuous AD probability as a binary predictor (Li, 2024). Such differences should be considered when interpreting model performance.

Several limitations of this study should be acknowledged. The relatively small sample size (*n* = 38) represents an important constraint. A *post-hoc* power analysis indicates that with 38 subjects and an assumed moderate correlation (*r* = 0.50) between observers, the study is powered at 80% (α = 0.05) to detect a difference in AUC of 0.21. While sufficient to identify substantial differences in discriminatory performance, statistical power for more subtle agreement metrics and correlation coefficients is sensitive to sampling variability. As a result, the findings should be interpreted as exploratory. Larger, independent cohorts will be required to confirm the observed patterns and improve generalizability. In addition, although cognitiveTree is designed for multi-class classification of neurodegenerative diseases, the present evaluation focused on CN-AD discrimination. This mismatch may lead to an underestimation of performance when alternative dementia categories receive the highest probability. Accordingly, the results should be interpreted within the context of this simplified evaluation framework. Radiologist assessments were obtained using probability assignments rather than routine clinical reporting, which may influence generalizability. Furthermore, the analysis was limited to structural MRI and did not incorporate clinical, neuropsychological, or biomarker data, which are often used in conjunction with imaging for dementia diagnosis. As cognitiveTree is an externally developed tool (Coupé et al., 2025), its internal architecture and training hyperparameters could not be independently verified or modified in the present study; this is an inherent constraint of validation studies using published automated pipelines. Finally, although cognitiveTree generates maps of brain structures deviating from normative aging patterns as a built-in output (Coupé et al., 2020), these deviation maps were not analyzed in the present study. The contribution of specific brain regions to classification decisions remains a key area for future research, where explainable AI (XAI) frameworks could help translate automated outputs into clinically interpretable anatomical patterns (Viswan et al., 2024). Explainability analysis incorporating regional contribution profiling represents an important direction for future work and would substantially strengthen the interpretability of cognitiveTree outputs in clinical contexts.

## 6 Conclusion

In this study, we systematically evaluated the diagnostic performance of an automated, multi-class neuroimaging classifier and expert radiologists for differentiating CN from AD using structural brain MRI. By applying a tie-aware evaluation framework that preserves probabilistic uncertainty, we demonstrated distinct performance profiles and agreement patterns between automated and human assessments. These results underscore the importance of aligning evaluation strategies with the underlying design and intended use of automated diagnostic tools. Rather than replacing human expertise, such systems may support diagnostic decision-making, particularly in complex or uncertain cases. Further validation and integration with clinical workflows will be important to determine their practical utility.

## Data Availability

Publicly available datasets were analyzed in this study. This data can be found here: https://adni.loni.usc.edu/data-samples/adni-data.
