# Correction: Reliability and diagnostic performance of an automated MRI-based classifier compared with radiologists in Alzheimer's disease

**DOI:** 10.3389/fninf.2026.1946431

**Published:** 2026-08-10

**Authors:** Nurmakhan Zholshybek, Elnora Abdurakhmanova, Almas Bimakhan, Dinara Jumadilova, Zhanibek Baiturlin, Joseph Almazan, Srinivasa Rao Bolla

**Affiliations:** 1 Department of Medicine, School of Medicine, Nazarbayev University, Astana, Kazakhstan; 2 Department of Biomedical Sciences, School of Medicine, Nazarbayev University, Astana, Kazakhstan; 3 Clinical and Academic Department of Radiology and Nuclear Medicine, Corporate Fund “University Medical Center”, Astana, Kazakhstan; 4 Department of Radiology and Radiosurgery, National Center for Neurosurgery, Astana, Kazakhstan

**Keywords:** Alzheimer's disease, artificial intelligence, brain MRI, cognitive disorders, diagnostic accuracy

In the published article, an incorrect **Funding** statement was provided. The sentence “This study was funded by a research grant of the Ministry of Science and Higher Education of the Republic of Kazakhstan (No. AP26199361) “Investigating the Relationship Between Sleep Quality and Brain Imaging Biomarkers” and Faculty Development grant (No. FDCRGP 2024-2026) of Nazarbayev University (No. SOM2024008) “Construction of Kazakh Brain Atlas and its role in the improvement of AI-based diagnostics of Dementia and Alzheimer's disease.” has been corrected to “This study was funded by a research grant of the Ministry of Science and Higher Education of the Republic of Kazakhstan (No. AP26199361) “Investigating the Relationship Between Sleep Quality and Brain Imaging Biomarkers” and Faculty Development Competitive Research Grant Program (FDCRGP) 2024–2026 of Nazarbayev University (No. 201223FD2605) “Construction of Kazakh Brain Atlas and its role in the improvement of AI-based diagnostics of Dementia and Alzheimer's disease.” The corrected **Funding** statement appears below.

“The author(s) declared that financial support was received for this work and/or its publication. This study was funded by a research grant of the Ministry of Science and Higher Education of the Republic of Kazakhstan (No. AP26199361) “Investigating the Relationship Between Sleep Quality and Brain Imaging Biomarkers” and Faculty Development Competitive Research Grant Program (FDCRGP) 2024–2026 of Nazarbayev University (No. 201223FD2605) “Construction of Kazakh Brain Atlas and its role in the improvement of AI-based diagnostics of Dementia and Alzheimer's disease.” The sponsors had no role in study design; in the collection, analysis, and interpretation of data; in the writing of the report; and in the decision to submit the paper for publication. Data collection and sharing for the Alzheimer's Disease Neuroimaging Initiative (ADNI) was funded by the National Institute on Aging (National Institutes of Health Grant U19AG024904). The grantee organization is the Northern California Institute for Research and Education. In the past, ADNI has also received funding from the National Institute of Biomedical Imaging and Bioengineering, the Canadian Institutes of Health Research, and private sector contributions through the Foundation for the National Institutes of Health (FNIH) including generous contributions from the following: AbbVie, Alzheimer's Association; Alzheimer's Drug Discovery Foundation; Araclon Biotech; BioClinica, Inc.; Biogen; BristolMyers Squibb Company; CereSpir, Inc.; Cogstate; Eisai Inc.; Elan Pharmaceuticals, Inc.; Eli Lilly and Company; EuroImmun; F. Hoffmann-La Roche Ltd and its affiliated company Genentech, Inc.; Fujirebio; GE Healthcare; IXICO Ltd.; Janssen Alzheimer Immunotherapy Research & Development, LLC.; Johnson & Johnson Pharmaceutical Research & Development LLC.; Lumosity; Lundbeck; Merck & Co., Inc.; Meso Scale Diagnostics, LLC.; NeuroRx Research; Neurotrack Technologies; Novartis Pharmaceuticals Corporation; Pfizer Inc.; Piramal Imaging; Servier; Takeda Pharmaceutical Company; and Transition Therapeutics.”

The original version of this article has been updated.

